# Bulk‐up synchronization of successive larval cohorts of *Anopheles gambiae* and *Anopheles coluzzii* through temperature reduction at early larval stages: effect on emergence rate, body size and mating success

**DOI:** 10.1186/s12936-021-03602-8

**Published:** 2021-02-02

**Authors:** Qaswa Zubair, Holly Matthews, Seynabou Sougoufara, Fatima Mujeeb, Simon Ashall, Fred Aboagye-Antwi, Frédéric Tripet

**Affiliations:** 1 Centre for Applied Entomology and Parasitology, School of Life Sciences, Keele University, Staffordshire, UK; 2 Department of Animal Biology and Conservation Science, School of Biological Sciences, College of Basic and Applied Sciences, University of Ghana, Legon-Accra, Ghana

**Keywords:** *Anopheles gambiae*, *Anopheles coluzzii*, Larvae development, Mass rearing, Mosquito release programmes, Mark release recapture studies

## Abstract

**Background:**

Malaria persists as a huge medical and economic burden. Although the number of cases and death rates have reduced in recent years, novel interventions are a necessity if such gains are to be maintained. Alternative methods to target mosquito vector populations that involve the release of large numbers genetically modified mosquitoes are in development. However, their successful introduction will require innovative strategies to bulk-up mosquito numbers and improve mass rearing protocols for *Anopheles* mosquitoes.

**Methods:**

The relationship between mosquito aquatic stage development and temperature was exploited so that multiple cohorts of mosquitoes, from separate egg batches, could be synchronized to ‘bulk-up’ the number of mosquitoes released. First instar larvae were separated into two cohorts: the first, maintained under standard insectary conditions at 27^o^C, the second subjected to an initial 5-day cooling period at 19^o^C.

**Results:**

Cooling of 1st instars slowed the mean emergence times of *Anopheles coluzzii* and *Anopheles gambiae* by 2.4 and 3.5 days, respectively, compared to their 27^o^C counterparts. Pupation and emergence rates were good (> 85 %) in all conditions. Temperature adjustment had no effect on mosquito sex ratio and adult fitness parameters such as body size and mating success.

**Conclusions:**

Bulk-up larval synchronization is a simple method allowing more operational flexibility in mosquito production towards mark-release-recapture studies and mass release interventions.

## Background

Malaria is a persistent public health issue. Despite over 50 years of sustained efforts to control the disease through the use of anti-malarial drugs and vector control, transmission has been interrupted in only a limited number of countries. The World Health Organization reported 228 million cases and 405,000 deaths in 2018 [1].

Most of these deaths occurred in children below five years of age living in sub-Saharan Africa. In recent years, the introduction of insecticide-treated bed nets (ITNs), long-lasting insecticidal nets (LLINs) and indoor residual spraying (IRS) combined with artemisinin-based combination therapy have resulted in a decline in malaria incidence, thus providing renewed hope for elimination goals [2, 3]. However, such gains are beginning to diminish, once again threatened by the development and spread of resistance to all anti-malarials and insecticides introduced [4, 5]. Furthermore, behavioural changes in mosquito vectors, such as biting at dawn or early evening rather than at night when people are under bed net protection, diminishes the effectiveness of current intra-domiciliary control measures [6–9]. Therefore, if reductions in malaria burden are to be at least sustained, alternative complementary approaches are necessary [10].

Following recent advances in genetic engineering, genetic vector control strategies for malaria mosquitos are now at the forefront of research and development goals [11–13]. These include a range of different approaches that are either self-limiting or self-sustaining. Self-limiting strategies involve the use of genetically modified sterile males or mosquitoes modified with a gene drive mechanism that is spatially or temporally self-limited [14, 15]. These methods bear similarities with the traditional sterile insect technique (SIT). Their impact depends on effective mating between released mosquitoes and the target population, and require repeated, inundative mass release of mosquitoes [16, 17]. Self-sustaining strategies employ a gene drive mechanism which means that a desirable trait such as male biased sex ratio [18, 19], reduced female fertility [20] or an antiparasitic effector gene [21] is inherited at a higher rate than mendelian inheritance. The spread of such self-propagating genes can lead to population suppression, reducing the number of biting females or population replacement with mosquitoes that are refractory to the malaria parasite [22, 23]. The self-sustaining strategies are a longer-term goal that would ideally require relatively smaller initial releases of mosquitoes, thereby making them more cost efficient [13, 15, 16]. However, the deployment of such genetic tools on a broader scale will still necessitate the production and release of much larger numbers of mosquitoes [13, 15, 16]. In addition to mosquito release interventions per se, ecological studies that focus on mosquito survival, dispersal or estimation of population sizes, such as mark-release-recapture studies, also rely on the punctual release of mosquitoes reared at a much smaller scale [24].

One major challenge in rearing *Anopheline* mosquitoes for release studies and interventions is that their eggs hatch shortly after being laid and can only survive for a limited number of days without water, hence, egg-to-adult rearing needs to be continuous [25]. This imposes constraints on rearing protocols and infrastructures and means that the release cohort largely depends on the number of adults in the preceding generation. There have been efforts towards the optimization of *Anopheline* egg storage. Through elaborate drying and cooling methods, it is now possible to increase egg storage times by up to 4–6 days, however beyond that point, hatch rate and larval development are negatively impacted [26–28]. Therefore, other avenues to bulk-up *Anopheles* mosquitoes for mass release, without affecting their phenotypic quality, should be explored.

The development rate of insects is mainly temperature dependent and offers the potential opportunity to slow or accelerate development [29]. In *Anopheles gambiae*, the relationship between mosquito aquatic stage development and temperature has been well studied [30–34]. Within a minimum and maximum threshold, development rate increases linearly with an increase in temperature. Indeed, Barreaux et al. [31] reported a 1.4-day difference in time to pupation between larvae maintained at 21^o^C and 29^o^C. Similarly, Christiansen-Jucht and colleagues reported a linear increase in development rate from 23 to 31 ^o^C, but at 35^o^C all larvae died before emergence [34]. Bayoh and Lindsay [32] showed that development rate increased linearly with temperatures from 22^o^C to 28^o^C resulting in an approximate 10-day shift in egg to adult development time. No adults emerged at temperatures below 18 ^o^C or above 34 ^o^C.

This study, aimed to exploit this relationship to mimic synchronization of successive egg batches obtained from repeated blood-feeding of a single female cohort, without impacting negatively on mosquito survival. The rearing temperature of 1st instar larvae of *An. gambiae sensu stricto* (*s.s*.) and *Anopheles coluzzii* laboratory strains was reduced with the aim of slowing down development by approximately 3 days, the time required for one gonotrophic cycle by females at 27 ^o^C [35–37]. The impact of the temperature alteration on the pupation and emergence rates, developmental times, adult phenotypic quality and mating success was evaluated. The ability to slow down a larval cohort by 3 days, hence to synchronize the emergence of adult progeny resulting from multiple blood feeds and successive egg batches from the same pool of females, has important implications for the optimization of mass production and release methods for *Anopheles sensu lato* (*s.l*.).

## Methods

### Mosquito maintenance

*Anopheles gambiae s.s*. Kisumu strain (an old strain colonized originally from Kisumu, Kenya, East Africa), and *Anopheles coluzzii*, VK3 strain (a strain colonized in 2018 from Vallée du Khou, Burkina Faso, West Africa), were maintained in the Manson Insectaries at the Centre for Applied Entomology and Parasitology, Keele University, UK. The strains were kept under standard, Manson insectary conditions: 27 ± 2^o^C, 12/12-hour light/dark cycle at 70 ± 5 % relative humidity unless otherwise stated [38]. Adults had a constant supply of 10 % glucose and were blood fed on defibrinated (fibrin removed to prevent clotting) horse blood (TCS Biosciences) using the Hemotek membrane feeding system (Blackburn, United Kingdom). Polystyrene cups, lined with Whatman filter paper, containing 50:50 deionized: mineral water was provided for oviposition. After hatching, 200 first instar larvae / 500ml mixed water (250ml deionized water + 250ml mineral water) were placed in trays (34 cm x 24 cm) and supplemented with 2 drops of Liquifry. Feeding with solid food commenced after 24 h, and all trays were provided with an additional 500ml water on day 5. Larvae were fed with an optimized feeding regime using ground TetraMin fish food (Tetramin, Tetra, Melle, Germany) and transferred to adult cages (5l plastic, 20.5 cm height x 20 cm diameter), upon pupation as described elsewhere [38, 39].

### Manipulation of larval temperature

**Fig. 1:**
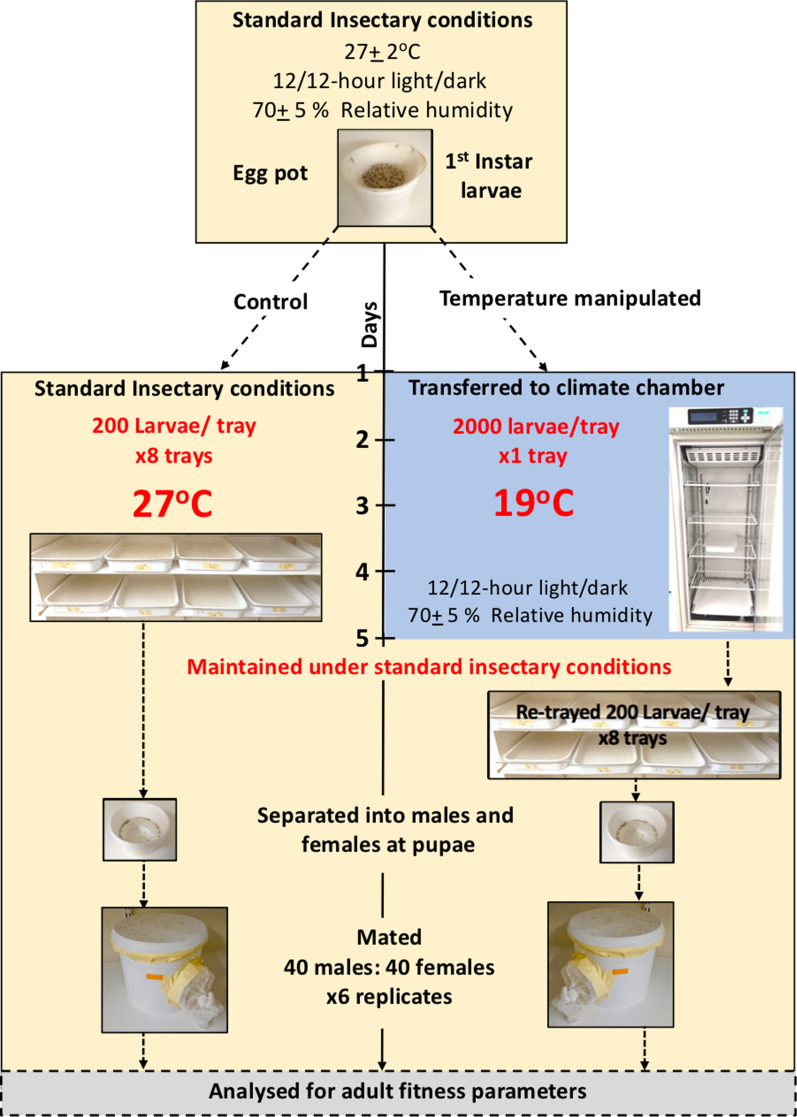
The experimental design. The workflow from 1st Instar to Adult emergence and mating is shown, including, the asymmetrical design of the study and the 5-day period of temperature manipulation

For each strain, 1st instar larvae from one egg batch were split into two groups: control and temperature manipulated (Fig. 1). The larvae in the control group were trayed in accordance with standard insectary protocol as described above (200 larvae/tray), 8 trays in total. The larvae in the temperature manipulated group were trayed at 2000 larvae/tray and placed in a climate chamber at 19^o^C 12/12-hour light/dark cycle at 70 ± 5 % relative humidity (Panasonic MLR Climatic Test Chambers 352H-PE Kadoma, Osaka, Japan). The temperature manipulated larvae remained at 19^o^C for 5 days and fed, first with Liquifry (as described previously), and then *ad libitum* with ground TetraMin fish food. On day 5, larvae kept at 19^o^C were transferred to standard insectary conditions (27 ± 2^o^C) and re-trayed at 200 larvae/tray (500ml of mixed water was added, with an additional 500ml of tap water). There were 8 trays in total. Larvae in the 27^o^C control group were reared according to the standard insectary protocol for the duration of the experiment.

### Adult development and mating

Adult emergence and pupae failing to emerge were recorded daily, as well as dead adults. For each experimental group, pupae were collected each day, sexed and placed into separate cages for males and females. Male and female mosquitoes aged 3–5 days old were combined (40 males + 40 females) into mating cages (6 cages total) and allowed to mate overnight. The following morning, mosquitoes were transferred to -20^o^C and stored in 75 % ethanol. Spermathecae from female mosquitoes were dissected and burst open in a drop of water. The presence of a coagulated sperm bundle provided confirmation of a successful mating event. Wing length was recorded for all females and a subsample of 15–30 males/condition as a proxy for adult size. In brief, a binocular microscope, calibrated using a stage micrometre (1mm = 10 eye piece units at x1 magnification) was used to measure one wing from each adult. Wings were measured from the distil end of the allula to the apical margin (radius veins) as described previously [40].

### Statistical analysis

Binomial variables such as pupation rates, emergence rates, sex ratio, and insemination rates were analysed *via* logistic regression. Emergence times were analysed *via* proportional hazard analysis. Likelihood odds ratios were used for *post-hoc* comparisons following logistic regression and proportional hazard analysis. Continuous data, such as wing length, (body size) was checked for normality and parametric and non-parametric tests were used where appropriate. In all multivariate analyses, interactions between independent variables were tested but removed from models if not significant. All analyses were carried out using the JMP 14 statistical software (SAS Institute, North Carolina).

## Results

### Effect of temperature manipulation on pupation and emergence rates

**Fig. 2:**
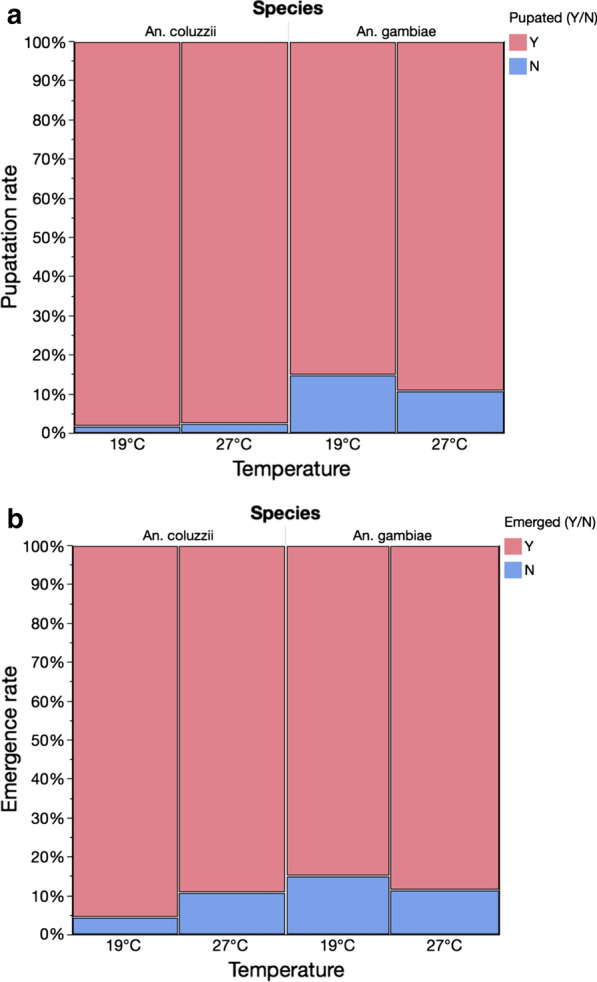
The effect of temperature manipulation on the pupation (**a**), and emergence (**b**) rates in ***Anopheles coluzzii*** and ***Anopheles gambiae***

Logistic regression analysis indicated that the reduction in temperature, to 19^o^C, during early larval development had no overall impact on pupation rates of *An. coluzzii* or *An. gambiae* (Likelihood ratio Chi-square = 3.63, *df* = 1 *P* = 0.057). There was a significant difference in pupation rates between the two species (LR = 144.23, *df* = 1, *P* < 0.001). Higher pupation rates were observed for *An. coluzzii* at both 19^o^C (98 %) and 27^o^C (97.5 %), however, *An. gambiae* also achieved high pupation rates at both temperatures; 85 % (19^o^C) and 89 % (27^o^C) (Fig. 2a).

High overall emergence rates (> 85 %) were observed for both species, however the effect of temperature depended on species (Table 1). Indeed, higher emergence rates were observed for *An. coluzzii* at 19^o^C, whereas for *An. gambiae* emergence was higher at 27^o^C (Fig. 2b).

**Table 1:** Logistic regression (effect likelihood ratio tests) of the effect of temperature and species on emergence rates

Source	DF	LR ChiSquare	Prob > ChiSq
Species	1	30.61	< 0.001
Temperature	1	5.79	0.016
Temperature*Species	1	24.15	< 0.001

DF Degrees of freedom, LR Likelihood Ratio

### Effect of temperature manipulation on sex ratio

None of the *An. coluzzii* and *An. gambiae* treatment groups differed from a male:female ratio of 1:1. For *An. coluzzii* at 19^o^C the proportion of males was 0.52 (95 % CI 0.48–0.56) and at 27^o^C the proportion of males was 0.54 (CI 0.50–0.58). For *An. gambiae* the male:female ratio for both temperatures was 0.51:0.49 (CIs +/- 0.04). Logistic regression analysis indicated that sex-ratios did not significantly differ between species (LR Chi-square = 2.14, *df* = 1, *P* = 0.144) nor by temperature condition (LR Chi-square = 0.002, *df* = 1, *P* = 0.965).

### Effect of temperature manipulation on emergence times

**Fig. 3:**
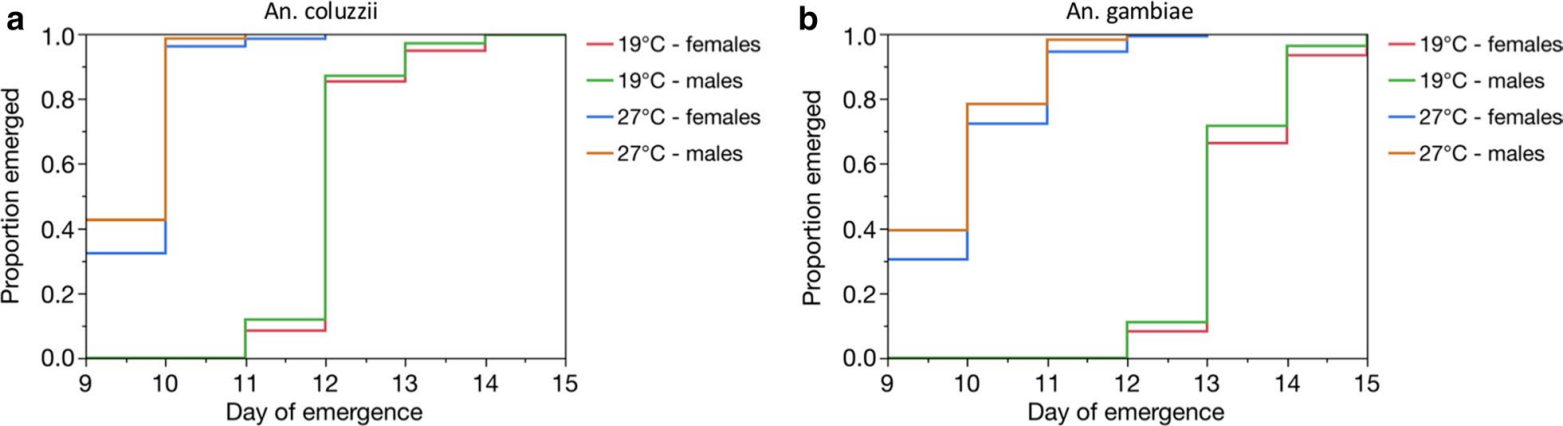
The effect of temperature manipulation on the emergence time of male and female ***Anopheles coluzzii*** (**a**) and ***Anopheles gambiae*** (**b**)

Proportional hazards analysis revealed that the emergence time of both *An. coluzzii* and *An. gambiae* was significantly affected by the 5-day cooling period (Table 2). *Anopheles coluzzii* took on average 2.4 and *An. gambiae* 3.5 days longer to emerge compared with those maintained at 27^o^C (Fig. 3). There were also significant differences in emergence times between species and sex. The interactions between species, sex and temperature were also found to be significant (Table 2).

**Table 2:** Logistic regression (effect likelihood ratio tests) for the effect of species, temperature and sex on emergence time

Source	DF	LR ChiSquare	Prob > ChiSq
Species	1	259.71	< 0.001
Temperature	1	2025.21	< 0.001
Sex	1	19.51	< 0.001
Temperature*Species	1	46.01	< 0.001
Temperature*Sex	1	5.60	0.018

### Effect of temperature manipulation on adult fitness parameters

**Fig. 4:**
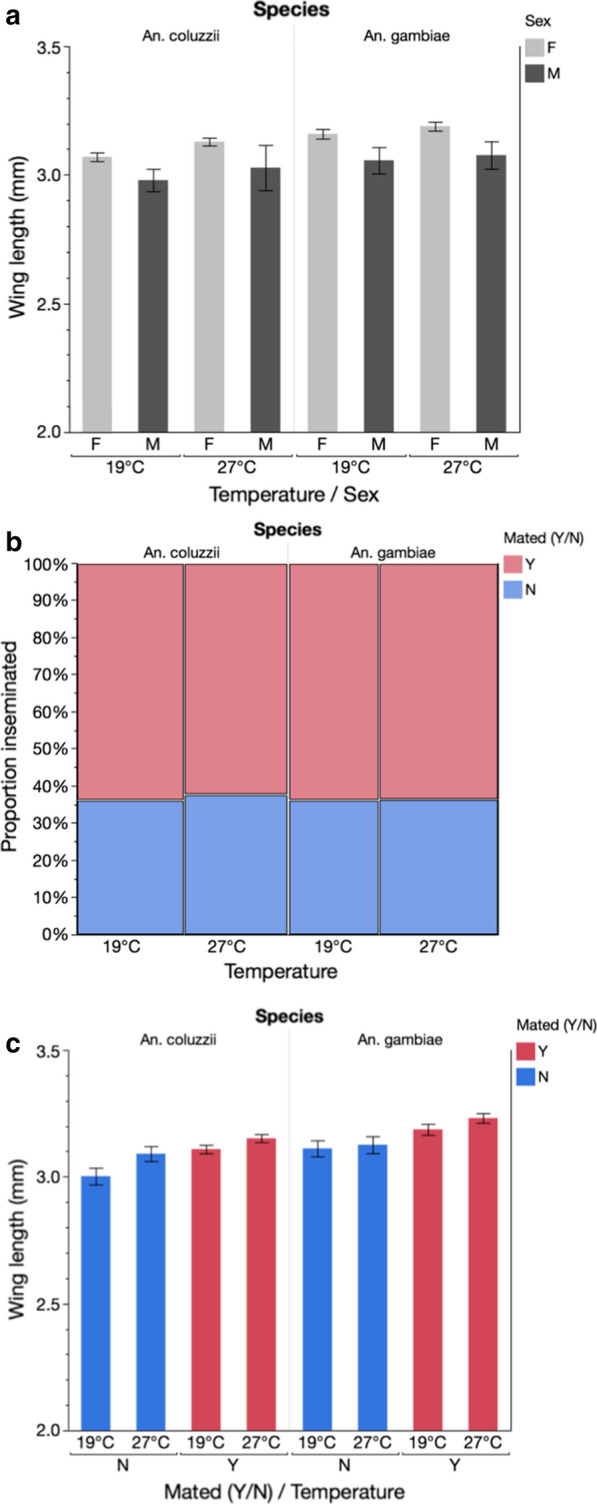
The effect of temperature manipulation on adult fitness parameters. **a** The effect of temperature on adult size of male and female *An. coluzzii* and *An. gambiae* mosquitoes. **b** The effect of temperature on mating status of *An. coluzzii* and *An. gambiae* females. **c** The effect of temperature on mating status with regard to mosquito size. Error bars represent 95 % confidence intervals

Multivariate analysis showed that mosquito wing length was significantly affected by species, temperature and sex (Table 3). *Anopheles coluzzii* individuals were significantly smaller than *An. gambiae* and male mosquitoes significantly smaller than females. Those exposed to the 19^o^C 5-day cooling period were significantly smaller than their counterparts maintained at 27^o^C (Table 3; Fig. 4a).

**Table 3:** General linear model effect data for the effect of species, temperature and sex on mosquito wing lengths

Source	DF	Sum of Squares	F Ratio	Prob > F
Species	1	1.20	70.86	< 0.001
Temperature	1	0.51	30.29	< 0.001
Sex	1	0.89	52.09	< 0.001

Insemination rates were similar at both temperatures for both species (Fig. 4b). When female size was also considered, differences were apparent between species, between temperatures and at different wing lengths (Table 4). Overall, inseminated females were larger in all conditions (Fig. 4c).

**Table 4:** Logistic regression (effect likelihood ratio tests) for the effect of species, temperature and wing length on mosquito inseminations rates

Source	DF	L-R ChiSquare	Prob > ChiSq
Species	1	6.63	0.010
Temperature	1	4.16	0.041
Wing length (mm)	1	94.00	< 0.001

## Discussion

The results showed that through temperature manipulation it is possible to delay emergence of mosquitoes by up to 3 days; the approximate length of the gonotrophic cycle of *Anopheline* females. These finding are important for ecological studies that require small punctual releases and for interventions requiring mass releases focussing on *Anopheline* vector species. Currently the logistics and planning for *Anopheline* production revolve around the assumption that achieved mosquito numbers, at a particular time point, directly depend on the quantity of eggs produced by a single gonotrophic cycle. The findings of this study offer the potential to effectively double the progeny produced from one female cohort, thereby bringing much needed flexibility to *Anopheline* rearing practices.

The 3-day delay was achieved by subjecting first instar larvae to a 5-day cooling period at 19^o^C. The alteration in temperature had no effect on pupation rates although there was a difference in the rate of pupation between *An. coluzzii* and *An. gambiae*. It was also found that cooling had a minimal effect on emergence rates, that were ≥ 85 %, but affected the two species conversely. In *An. coluzzii*, it resulted in an increase in emergence rate, but in *An. gambiae* it resulted in small decrease in emergence rate. Overall, pupation and emergence rates were high and in line with reports elsewhere for laboratory-reared *Anopheles* [41, 42].

There was no effect of temperature reduction on sex ratio, which was equivalent to a 1:1 male to female ratio in both species. Any evidence of female bias would have important consequences for male-focused mass release programmes. Imbalances have been reported following temperature and diet alterations for *Aedes* mosquitoes [43, 44]. However, for *Anopheles* mosquitoes no such differences have been found [45, 46].

Adult phenotypic quality and mating competitiveness are crucial to the success of release programmes [39, 47, 48]. Several studies have reported negative carry-over effects on the phenotypic quality of adult mosquitoes following experimental manipulations of larval conditions, such as temperature, density and food availability [31, 38, 49]. This study found that male and female adults reared at 19^o^C were smaller than those reared at 27^o^C, but the 0.05 mm (1.5 %) reduction in size observed was unlikely to be biologically important. Indeed, the negligible size differences found did not translate to a negative impact on insemination rates. In the natural setting, *An. gambiae s.l.* mate in swarms that are typically composed of males and females which visit to choose a mate and then leave *in copula* [17, 50]. Smaller males have reduced spermatogenesis and are less competitive in terms of mating than medium-to-large sized mosquitoes, making them poor candidates for release programmes [51, 52]. Compared to the size distribution from those reports (2.48–3.12), males produced in this study at either temperature, were relatively large (2.98–3.08 mm) and consistent with the optimal size of 3mm for mating found in field studies [17].

Smaller females have reduced fecundity, are more likely to require multiple blood feeds before completion of a gonotophic cycle and may be less attractive to males [34, 39, 53, 54]. Although the current study found no difference in overall insemination rates in relation to larval cooling, inseminated *An. coluzzii* and *An. gambiae* females were 0.08 mm (2.7 %) and 0.09 mm (2.9 %) larger than non-inseminated ones, respectively. Although, this is again a very small size difference, the finding that larger females were more likely to mate is consistent with results from insectary and field swarm studies that suggest males might prefer to mate with larger females [17].

The current study opted to slow down larval development rate by lowering the temperature rather than speed it up by increasing the temperature. Studies elsewhere have shown that at temperatures > 34^o^C there are negative, irreversible carry-over effects on surviving adult mosquitoes and overall survival is lower [32, 34, 55]. Indeed, although adults develop quicker, they are smaller [31, 34, 56] possibly because food consumption cannot sustain the rate of metabolism [57]. Therefore, the current study corroborated previous reports which found that cooling temperatures serve as a reversible inhibitor to mosquito development with negligible impacts on mosquito phenotypic quality, provided they are not maintained throughout their entire development [32, 58]. A relatively short 5-day cooling period of 1st instars was employed, which allowed rearing at 10-fold higher density and *ad libitum* feeding. In preliminary studies, attempts to also maintain 1st instar larvae at comparable densities at 27^o^C, found that larval competition negatively affected development rates and success. Hence, keeping 1st instars at high densities was only possible for larvae kept at a cooler temperature which reduced their metabolism and food consumption [57, 59]. The optimized protocol presented here, therefore, exploits the relationship between development rate, temperature, density and food availability to adjust emergence time by appromimately 3 days. As an incubator/fridge will be required for the cooled temperature condition, the 10-fold higher density culture at 19^o^C make the method both practical and scalable whilst minimizing pressure in terms of insectary space.

## Conclusions

In conclusion, this study presents an optimized translatable methodology to increase *Anopheles* numbers for release studies and programmes. The optimized regime including a 5-day reduction in temperature (from 27^o^C to 19^o^C), adapted feeding and increased density represents a practical and scalable addition to mosquito production protocols. Here a 2.4 and 3.5-day delay was achieved for *An. coluzzii* and *An. gambiae* emergence times, respectively with no or negligible impacts on mosquito numbers, adult body size and mating rates. Using 18^o^C to slowdown larval development will ensure that a 3-day delay is achieved under all circumstances. As the 3-day delay spans the duration of one gonotrophic cycle the inclusion of a cooling period into mosquito mass rearing protocols offers the potential to synchronize successive larvae batches from a single pool of females. This is a modest but much needed step towards the optimization of rearing techniques geared specifically for *Anopheles* mosquitoes, one of the most important groups of disease vectors.

## Data Availability

All data generated or analysed during this study are included in this published article.

## References

[1] WHO Global Malaria Programme. World malaria report 2019. Geneva: World Health Organization; 2019.

[2] Lengeler C (2004). Insecticide-treated nets for malaria control: real gains. Bull World Health Organ.

[3] Bhatt S, Weiss DJ, Cameron E, Bisanzio D, Mappin B, Dalrymple U (2015). The effect of malaria control on *Plasmodium falciparum* in Africa between 2000 and 2015. Nature.

[4] Ranson H, N’Guessan R, Lines J, Moiroux N, Nkuni Z, Corbel V (2011). Pyrethroid resistance in African anopheline mosquitoes: what are the implications for malaria control?. Trends Parasitol.

[5] Conrad MD, Rosenthal PJ (2019). Antimalarial drug resistance in Africa: the calm before the storm?. Lancet Infect Dis.

[6] Dambach P, Schleicher M, Korir P, Ouedraogo S, Dambach J, Sié A (2018). Nightly biting cycles of *Anopheles* species in rural Northwestern Burkina Faso. J Med Entomol.

[7] Gatton ML, Chitnis N, Churcher T, Donnelly MJ, Ghani AC, Godfray HC (2013). The importance of mosquito behavioural adaptations to malaria control in Africa. Evolution.

[8] Russell TL, Govella NJ, Azizi S, Drakeley CJ, Kachur SP, Killeen GF (2011). Increased proportions of outdoor feeding among residual malaria vector populations following increased use of insecticide-treated nets in rural Tanzania. Malar J.

[9] Sougoufara S, Diédhiou SM, Doucouré S, Diagne N, Sembène PM, Harry M (2014). Biting by *Anopheles funestus* in broad daylight after use of long-lasting insecticidal nets: a new challenge to malaria elimination. Malar J.

[10] Sougoufara S, Ottih EC, Tripet F (2020). The need for new vector control approaches targeting outdoor biting Anopheline malaria vector communities. Parasit Vectors.

[11] Alphey L, Beard CB, Billingsley P, Coetzee M, Crisanti A, Curtis C (2002). Malaria control with genetically manipulated insect vectors. Science.

[12] Marshall JM, Taylor CE (2009). Malaria control with transgenic mosquitoes. PLoS Med.

[13] Hammond AM, Galizi R (2017). Gene drives to fight malaria: current state and future directions. Pathog Glob Health.

[14] Dhole S, Vella MR, Lloyd AL, Gould F (2018). Invasion and migration of spatially self-limiting gene drives: a comparative analysis. Evol Appl.

[15] Burt A, Deredec A (2018). Self-limiting population genetic control with sex-linked genome editors. Proc Biol Sci.

[16] Burt A (2014). Heritable strategies for controlling insect vectors of disease. Philos Trans R Soc Lond B Biol Sci.

[17] Diabate A, Tripet F (2015). Targeting male mosquito mating behaviour for malaria control. Parasit Vectors.

[18] Kyrou K, Hammond AM, Galizi R, Kranjc N, Burt A, Beaghton AK (2018). A CRISPR-Cas9 gene drive targeting doublesex causes complete population suppression in caged *Anopheles gambiae* mosquitoes. Nat Biotechnol.

[19] Simoni A, Hammond AM, Beaghton AK, Galizi R, Taxiarchi C, Kyrou K (2020). A male-biased sex-distorter gene drive for the human malaria vector *Anopheles gambiae*. Nat Biotechnol.

[20] Hammond A, Galizi R, Kyrou K, Simoni A, Siniscalchi C, Katsanos D (2016). A CRISPR-Cas9 gene drive system targeting female reproduction in the malaria mosquito vector *Anopheles gambiae*. Nat Biotechnol.

[21] Ito J, Ghosh A, Moreira LA, Wimmer EA, Jacobs-Lorena M (2002). Transgenic anopheline mosquitoes impaired in transmission of a malaria parasite. Nature.

[22] Beaghton A, Hammond A, Nolan T, Crisanti A, Godfray HC, Burt A (2017). Requirements for driving antipathogen effector genes into populations of disease vectors by homing. Genetics.

[23] James AA (2005). Gene drive systems in mosquitoes: rules of the road. Trends Parasitol.

[24] Epopa PS, Millogo AA, Collins CM, North A, Tripet F, Benedict MQ (2017). The use of sequential mark-release-recapture experiments to estimate population size, survival and dispersal of male mosquitoes of the *Anopheles gambiae* complex in Bana, a west African humid savannah village. Parasit Vectors.

[25] Clements AN (1992). The biology of mosquitoes.

[26] Khan I, Damiens D, Soliban SM, Gilles JR (2013). Effects of drying eggs and egg storage on hatchability and development of *Anopheles arabiensis*. Malar J.

[27] Mazigo E, Kidima W, Myamba J, Kweka EJ (2019). The impact of *Anopheles gambiae* egg storage for mass rearing and production success. Malar J.

[28] Lobb L, Munhenga G, Yamada H, Koekemoer L (2019). The effect of egg storage of laboratory reared *Anopheles arabiensis* (Diptera: Culicidae) on egg hatch synchronisation, pupation success and pupal production time. Afr Entomol.

[29] Ratte HT, Hoffmann KH (1985). Temperature and Insect Development. Environmental Physiology and Biochemistry of Insects.

[30] Armstrong JA, Bransby-Williams WR (1961). The maintenance of a colony of *Anopheles gambiae*, with observations on the effects of changes in temperature. Bull World Health Organ.

[31] Barreaux AMG, Stone CM, Barreaux P, Koella JC (2018). The relationship between size and longevity of the malaria vector *Anopheles gambiae* (s.s.) depends on the larval environment. Parasit Vectors.

[32] Bayoh MN, Lindsay SW (2003). Effect of temperature on the development of the aquatic stages of *Anopheles gambiae sensu stricto* (Diptera: Culicidae). Bull Entomol Res.

[33] Beck-Johnson LM, Nelson WA, Paaijmans KP, Read AF, Thomas MB, Bjørnstad ON (2013). The effect of temperature on *Anopheles* mosquito population dynamics and the potential for malaria transmission. PLoS One.

[34] Christiansen-Jucht CD, Parham PE, Saddler A, Koella JC, Basáñez MG (2015). Larval and adult environmental temperatures influence the adult reproductive traits of *Anopheles gambiae* s.s. Parasit Vectors.

[35] Lardeux FJ, Tejerina RH, Quispe V, Chavez TK (2008). A physiological time analysis of the duration of the gonotrophic cycle of *Anopheles pseudopunctipennis* and its implications for malaria transmission in Bolivia. Malar J.

[36] Mala AO, Irungu LW, Mitaki EK, Shililu JI, Mbogo CM, Njagi JK (2014). Gonotrophic cycle duration, fecundity and parity of *Anopheles gambiae* complex mosquitoes during an extended period of dry weather in a semi arid area in Baringo County, Kenya. Int J Mosq Res.

[37] Reisen WK, Aslamkhan M (1979). A release-recapture experiment with the malaria vector, *Anopheles stephensi* Liston, with observations on dispersal, survivorship, population size, gonotrophic rhythm and mating behaviour. Ann Trop Med Parasitol.

[38] Aboagye-Antwi F, Tripet F (2010). Effects of larval growth condition and water availability on desiccation resistance and its physiological basis in adult *Anopheles gambiae sensu stricto*. Malar J.

[39] Ekechukwu NE, Baeshen R, Traorè SF, Coulibaly M, Diabate A, Catteruccia F, et al. Heterosis increases fertility, fecundity, and survival of laboratory-produced F1 hybrid males of the malaria mosquito *Anopheles coluzzii*. G3 (Bethesda). 2015;5:2693 – 709. 10.1534/g3.115.021436 PMC4683642 26497140

[40] Akpodiete NO, Diabate A, Tripet F (2019). Effect of water source and feed regime on development and phenotypic quality in *Anopheles gambiae* (s.l.): prospects for improved mass-rearing techniques towards release programmes. Parasit Vectors.

[41] Kweka EJ, Tenu F, Magogo F, Mboera LEG (2015). *Anopheles gambiae* sensu stricto aquatic stages development comparison between insectary and semifield structure. Adv Zool.

[42] Epopa PS, Maiga H, Hien DFS, Dabire RK, Lees RS, Giles J (2018). Assessment of the developmental success of *Anopheles coluzzii* larvae under different nutrient regimes: effects of diet quality, food amount and larval density. Malar J.

[43] Farjana T, Tuno N, Higa Y (2012). Effects of temperature and diet on development and interspecies competition in *Aedes aegypti* and *Aedes albopictus*. Med Vet Entomol.

[44] Sasmita HI, Tu WC, Bong LJ, Neoh KB (2019). Effects of larval diets and temperature regimes on life history traits, energy reserves and temperature tolerance of male *Aedes aegypti* (Diptera: Culicidae): optimizing rearing techniques for the sterile insect programmes. Parasit Vectors.

[45] Kirby MJ, Lindsay SW (2009). Effect of temperature and inter-specific competition on the development and survival of *Anopheles gambiae* sensu stricto and *An. arabiensis* larvae. Acta Trop.

[46] Phasomkusolsil S, Lerdthusnee K, Khuntirat B, Kongtak W, Pantuwatana K, Murphy JR (2011). Effect of temperature on laboratory reared *Anopheles dirus* Peyton and Harrison and *Anopheles sawadwongporni* Rattanarithikul and Green. Southeast Asian J Trop Med Public Health.

[47] Davidson G, Odetoyinbo JA, Colussa B, Coz J (1970). Field attempt to assess the mating competitiveness of sterile males produced by crossing 2 member species of the *Anopheles gambiae* complex. Bull World Health Organ.

[48] Smidler AL, Scott SN, Mameli E, Shaw WR, Catteruccia F (2018). A transgenic tool to assess *Anopheles* mating competitiveness in the field. Parasit Vectors.

[49] Ezeakacha NF, Yee DA (2019). The role of temperature in affecting carry-over effects and larval competition in the globally invasive mosquito *Aedes albopictus*. Parasit Vectors.

[50] Diabaté A, Yaro AS, Dao A, Diallo M, Huestis DL, Lehmann T (2011). Spatial distribution and male mating success of *Anopheles gambiae* swarms. BMC Evol Biol.

[51] Maïga H, Niang A, Sawadogo SP, Dabiré RK, Lees RS, Gilles JR (2014). Role of nutritional reserves and body size in *Anopheles gambiae* males mating success. Acta Trop..

[52] Ng’habi KR, Huho BJ, Nkwengulila G, Killeen GF, Knols BGJ, Ferguson HM (2008). Sexual selection in mosquito swarms: may the best man lose?. Anim Behav.

[53] Okanda FM, Dao A, Njiru BN, Arija J, Akelo HA, Touré Y (2002). Behavioural determinants of gene flow in malaria vector populations: *Anopheles gambiae* males select large females as mates. Malar J.

[54] De Jesus CE, Reiskind MH (2016). The importance of male body size on sperm uptake and usage, and female fecundity in *Aedes aegypti* and *Aedes albopictus*. Parasit Vectors.

[55] Zapletal J, Erraguntla M, Adelman ZN, Myles KM, Lawley MA (2018). Impacts of diurnal temperature and larval density on aquatic development of *Aedes aegypti*. PLoS One.

[56] Thailayil J, Magnusson K, Godfray HC, Crisanti A, Catteruccia F (2011). Spermless males elicit large-scale female responses to mating in the malaria mosquito *Anopheles gambiae*. Proc Natl Acad Sci USA.

[57] Korochkina SE, Gordadze AV, Zakharkin SO, Benes H (1997). Differential accumulation and tissue distribution of mosquito hexamerins during metamorphosis. Insect Biochem Mol Biol.

[58] Jalil M (1972). Effect of temperature on larval growth of *Aedes triseriatus*. J Econ Entomol.

[59] Hood-Nowotny R, Schwarzinger B, Schwarzinger C, Soliban S, Madakacherry O, Aigner M (2012). An analysis of diet quality, how it controls fatty acid profiles, isotope signatures and stoichiometry in the malaria mosquito *Anopheles arabiensis*. PLoS ONE.

